# Shape matters: unsupervised exploration of IDH-wildtype glioma imaging survival predictors

**DOI:** 10.1007/s00330-024-11042-6

**Published:** 2024-09-09

**Authors:** Martha Foltyn-Dumitru, Mustafa Ahmed Mahmutoglu, Gianluca Brugnara, Tobias Kessler, Felix Sahm, Wolfgang Wick, Sabine Heiland, Martin Bendszus, Philipp Vollmuth, Marianne Schell

**Affiliations:** 1 Department of Neuroradiology, Heidelberg University Hospital, Heidelberg, Germany; 2 Section for Computational Neuroimaging, Department of Neuroradiology, Heidelberg University Hospital, Heidelberg, Germany; 3 Department of Neurology and Neurooncology Program, Heidelberg University Hospital, Heidelberg University, Heidelberg, Germany; 4 Clinical Cooperation Unit Neurooncology, German Cancer Research Center (DKFZ), Heidelberg, Germany; 5 Department of Neuropathology, Heidelberg University Hospital, Heidelberg, Germany; 6 Clinical Cooperation Unit Neuropathology, German Cancer Consortium (DKTK), German Cancer Research Center (DKFZ), Heidelberg, Germany; 7 Department of Neuroradiology, Bonn University Hospital, Bonn, Germany

**Keywords:** Magnetic resonance imaging, Glioma, Radiomics, Cluster analysis

## Abstract

**Objectives:**

This study examines clustering based on shape radiomic features and tumor volume to identify IDH-wildtype glioma phenotypes and assess their impact on overall survival (OS).

**Materials and methods:**

This retrospective study included 436 consecutive patients diagnosed with IDH-wt glioma who underwent preoperative MR imaging. Alongside the total tumor volume, nine distinct shape radiomic features were extracted using the PyRadiomics framework. Different imaging phenotypes were identified using partition around medoids (PAM) clustering on the training dataset (348/436). The prognostic efficacy of these phenotypes in predicting OS was evaluated on the test dataset (88/436). External validation was performed using the public UCSF glioma dataset (*n* = 397). A decision-tree algorithm was employed to determine the relevance of features associated with cluster affiliation.

**Results:**

PAM clustering identified two clusters in the training dataset: Cluster 1 (*n* = 233) had a higher proportion of patients with higher sphericity and elongation, while Cluster 2 (*n* = 115) had a higher proportion of patients with higher maximum 3D diameter, surface area, axis lengths, and tumor volume (*p* < 0.001 for each). OS differed significantly between clusters: Cluster 1 showed a median OS of 23.8 compared to 11.4 months of Cluster 2 in the holdout test dataset (*p* = 0.002). Multivariate Cox regression showed improved performance with cluster affiliation over clinical data alone (*C* index 0.67 vs 0.59, *p* = 0.003). Cluster-based models outperformed the models with tumor volume alone (evidence ratio: 5.16–5.37).

**Conclusion:**

Data-driven clustering reveals imaging phenotypes, highlighting the improved prognostic power of combining shape-radiomics with tumor volume, thereby outperforming predictions based on tumor volume alone in high-grade glioma survival outcomes.

**Clinical relevance statement:**

Shape-radiomics and volume-based cluster analyses of preoperative MRI scans can reveal imaging phenotypes that improve the prediction of OS in patients with IDH-wild type gliomas, outperforming currently known models based on tumor size alone or clinical parameters.

**Key Points:**

- *Shape radiomics and tumor volume clustering in IDH-wildtype gliomas are investigated for enhanced prognostic accuracy*.
- *Two distinct phenotypic clusters were identified with different median OSs*.
- *Integrating shape radiomics and volume-based clustering enhances OS prediction in IDH-wildtype glioma patients*.

## Introduction

Isocitrate dehydrogenase-wildtype (IDH-wt) gliomas are aggressively malignant brain tumors, which pose significant challenges in clinical management with a dismal prognosis [1], necessitating advancements in diagnostic and therapeutic strategies [2, 3]. Accurate prediction of overall survival (OS) is crucial for devising effective treatment strategies for IDH-wt glioma patients. While RANO (response assessment in neuro-oncology) 2.0 criteria introduced tumor volume as a parameter for assessing therapy response [4]. While tumor volume is a valuable prognostic indicator, especially preoperatively [5, 6] its use alone has limitations. Specifically, it does not account for the heterogeneity of tumor morphology, which can provide critical insights into tumor behavior and patient prognosis.

Shape features such as the location of the tumor, its contour, and geometric properties in structural MRI are instrumental in qualitatively describing the tumor environment [7–9]. Notably, these features have been linked to the genetic phenotype of brain tumors [10] and predict OS in IDH-wt glioma patients [11–13]. Unlike non-shape features like high-order radiomics, shape features are highly reproducible and do not rely on intensity normalization procedures [14], demonstrating robustness against variations in image acquisition parameters and image noise. Whether acquired manually or extracted as quantitative high-throughput data from medical images, as seen in shape radiomics from radiological images [15], these features offer a quantitative approach to characterizing lesion topology. They can be categorized into global and local attributes. Global features focus on the lesion’s contour, providing measurements like roundness and perimeter, including diameters of major and minor axes. On the other hand, local shape features delve into the surface curvature aspects of isosurfaces, quantifying elements such as curvedness and sharpness [16].

Despite the potential of shape features, the clinical gold standard still relies heavily on measuring the tumor’s two-dimensional diameters through the imaging plane or (semi)automatic three-dimensional volumetric measurements [17, 18]. However, this approach overlooks the detailed shape characteristics that can offer additional prognostic information. The current performance of this standard is limited in capturing the full complexity of tumor heterogeneity.

This study aims to uncover distinct imaging phenotypes in newly diagnosed, untreated IDH-wt glioma patients by focusing on tumor volume and shape radiomic features. Utilizing unsupervised partition around medoids (PAM) clustering, we aim to identify these phenotypes, followed by the development of decision-tree models to elucidate the intrinsic relationship between imaging parameters and the identified phenotypes. Through this investigation, we seek to enrich the understanding of IDH-wt glioma heterogeneity and its implications for prognostic subtyping, leveraging the insights gained from tumor volume and shape radiomics.

## Materials and methods

### Dataset

This retrospective study received approval from the University of Heidelberg’s local ethics committee, with an exemption from the requirement of informed consent (S-784 2018). The analysis encompassed a series of consecutive patients (*n* = 439) diagnosed with IDH-wt glioma, as classified by the WHO 2021 criteria [19]. All patients underwent preoperative MRI at the Department of Neuroradiology, Heidelberg University Hospital, Germany between 2009 and 2020, with comprehensive clinical documentation maintained. DNA methylation assays provided the IDH status for all participants [20]. OS data were extracted from patient records. In cases where a death date was not recorded (due to ongoing survival or loss to follow-up), the date of the last known contact was noted, and the patient’s data were subsequently censored.

For analytical purposes, the dataset was bifurcated using tidymodels [21], allocating 80% of the data to a training set and the remaining 20% to a test set.

External testing was performed using the publicly available preoperative MRI glioma dataset of the University of California San Francisco (UCSF) with 501 patients [22]. As in the training dataset, data in the external validation included T1-weighted images before and after contrast administration, FLAIR, and T2-weighted images. All examinations were downloaded in NifTI (Neuroimaging Informatics Technology Initiative) format, information on the sequences included in the datasets is found at 10.7937/tcia.bdgf-8v37.

### MRI examinations and radiomics extraction

Imaging acquisition was conducted as part of standard clinical procedures using a 3-T MR system (comprising Magnetom Verio, TIM Trio, or Skyra models from Siemens Healthcare) equipped with a 12-channel head-matrix coil. Adhering to international imaging standards [23], the protocol included the acquisition of 3D T1-weighted images pre (T1) and post (cT1) administration of a 0.1 mmol/kg gadoterate meglumine bolus (Dotarem, Guerbet), along with axial 2D FLAIR and T2-weighted images.

The DICOM (Digital Imaging and Communications in Medicine) files were converted to NIfTI format using the freely available software dcm2nii (https://www.nitrc.org/plugins/mwiki/index.php/dcm2nii:MainPage) [24]. For tumor segmentation, a custom variant of the HD-GLIO tool, employing deep learning algorithms, was utilized to identify necrotic, contrast-enhanced, and non-contrast-enhanced T2/FLAIR hyperintense compartments within the tumors [25, 26]. These automated segmentations were reviewed and refined by M.F., a neuroradiology resident with six years of experience, ensuring accuracy and precision in the segmentation process.

Feature extraction and selection were performed in Python (version 3.8.5) using PyRadiomics (version 3.0.1, https://pyradiomics.readthedocs.io) [27]. For the feature extraction, the different segmentation masks of contrast-enhancing, noncontrast-enhanced T2/FLAIR hyperintense, and necrotic components of the tumor were combined into one segmentation mask, so the radiomics were extracted from the whole tumor. Nine, according to the Image Biomarker Standardization Initiative, reproducible shape radiomic features, namely maximum 3D diameter, major axis length, minor axis length, least axis length, surface area, sphericity, elongation, flatness, and surface volume ratio, were calculated on the T1w pre-contrast image [28]. A detailed description of the features can be found in Supplementary Materials and Methods and a comparison of the features between the HD and UCSF datasets is summarized in Supplementary Table 1. The whole tumor volume was extracted using FSL (FMRIB software library, FSL, http://fsl.fmrib.ox.ac.uk/fsl/fslwiki/FSL).

### Clustering

All the following analysis steps were performed on the training dataset only and later applied to the test dataset and UCSF dataset.

### Cut-off determination and distance calculation

Prognostic thresholds were determined for each radiomic feature and the whole tumor volume (*n* = 10). Since the calculation of the threshold requires a normal distribution, we applied a Box–Cox transformation to each parameter to approximate a normal distribution. This transformation was implemented using the recipes package in R (https://cran.r-project.org/web/packages/recipes/index.html). The lambda value for the Box–Cox transformation was saved for each parameter. Next, we used a hierarchical Bayesian method under the framework of the Cox proportional hazards model to fit each parameter for OS. This fitting was performed with the bhm package in R (https://cran.r-project.org/web/packages/bhm), using the default parameters with *B* = 500 burn-in steps and *R* = 2000 replications, as recommended by the package developers. Since the cut-off values were calculated on the Box–Cox transformed data, we transformed these cut-off values back to the original scale of the parameters using the stored lambda values. This reverse step ensured that the cut-off values were on the same scale as the original data. Only these back-transformed cut-off values were used for subsequent steps. All cut-off values and corresponding lambda values are summarized in Supplementary Table 2.

Based on these cut-off values, each parameter for each patient was binarized into either low (less than the cut-off value) or high (greater than the cut-off value). Subsequently, a distance matrix was calculated using the Gower distance for each patient, incorporating the ten binarized imaging parameters (nine radiomics features and the whole tumor volume). This approach allows for a comprehensive comparison of the imaging characteristics across the patient cohort.

### PAM clustering

The utilization of the PAM clustering methodology presented distinct advantages, notably its capacity to designate representative members within each cluster through medoids—central data points that significantly enhance the interpretability of results [29]. The method’s efficacy in handling categorical variables, including binary parameters like ours, further underscored its appropriateness for this analysis. Moreover, PAM clustering exhibited remarkable stability, a critical factor for ensuring the replicability of the cluster formations. In our implementation, medoids, selected as the central points of clusters, were operationalized using the ‘cluster’ package in R [30]. The determination of the optimal cluster count was guided by the computation of the average silhouette coefficient. To rigorously assess the stability of the clusters, we calculated the Jaccard index, utilizing 500 bootstrapping samples for a robust estimation. Subsequently, for the patients in the test dataset, Gower distances were computed concerning these calculated medoids. The assignment of patients to specific clusters was based on the proximity to the nearest medoid, with the smallest Gower distance being the determinant criterion for cluster membership.

### Explanatory analysis with tree-based modeling

To ascertain the impact of imaging parameters on cluster allocation, we constructed a decision-tree model utilizing the tidymodels library [21]. Optimization of hyperparameters was achieved through grid search, incorporating a 10-fold cross-validation that focused on optimizing tree depth and cost complexity to enhance model accuracy. Additionally, determining feature significance within the models was facilitated by employing variable importance plots, illustrating each feature’s relative importance in predicting the outcome (available at https://github.com/koalaverse/vip/).

### Statistical analysis

All data analyses were conducted using the R software (version 4.2.2, R Foundation for Statistical Computing). Detailed versions of these packages are provided in Supplementary Table 3. Continuous variables were expressed as means with standard deviations or medians with interquartile ranges, while categorical variables were presented as counts and frequencies. The Wilcoxon–Mann–Whitney test was employed for comparing continuous variables, and chi-squared tests were used for categorical variables.

Survival curves for OS in both clusters within the test and UCSF datasets were constructed using the Kaplan–Meier method, accompanied by the log-rank test for statistical assessment. Both univariate and multivariate Cox proportional hazards regression models were applied to evaluate OS concerning cluster affiliation and overall tumor volume, with adjustments made for potential clinical confounders including age, sex, and preoperative ECOG performance status.

The discriminative capability of the clusters was assessed through the calculation of the concordance probability (*C* index) and the Akaike information criterion (AIC) for each model [31]. Comparative analysis of embedded models was performed using analysis of variance focused on the *C* indices to determine statistical significance. Furthermore, differences between non-embedded models were evaluated by computing the evidence ratio based on the AIC values.

For all analyses in this study, a *p*-value threshold of less than 0.05 was established as the criterion for statistical significance. This study strictly adhered to the CheckList for EvaluAtion of Radiomics reporting guidelines to enhance the credibility, reproducibility, and transparency of the study [32]. The checklist is available in the Electronic Supplemental Material.

## Results

### Patient characteristics

In total, 439 patients with new IDH-wt glioma were initially screened, of which 436 met the inclusion criteria and were subsequently enrolled in the study. The exclusion of three patients occurred due to compromised MRI quality, notably marked by significant motion artifacts. Detailed demographic data and image characteristics of the study cohort are provided in Table 1. For analytical purposes, the cohort was segregated into training (*n* = 348) and test (*n* = 88) datasets. Kaplan–Meier survival analysis, as depicted in Supplementary Fig. 1, revealed no statistically significant difference in OS between these two datasets (log-rank test *p* = 0.17).

**Table 1:** Demographic and volume data of the HD and UCSF datasets as well as of the training and test split

Parameter		All datasets			HD dataset		
		HD	UCSF	*p*-value	Train	Test	*p*-value
Total no. of patients		436	397	NA	348	88	NA
Sex [*n* (%)]	Female	197 (45)	162 (41)	0.20	158 (45)	39 (44)	0.86
Age [y]	Mean ± std.	64 ± 12	62 ± 12	0.08	63 ± 11	63 ± 13	0.74
Tumor volume [cm^3^]	Mean ± std.	84 ± 59	91 ± 58	0.07	85 ± 59	83 ± 58	0.91

Uncorrected *p*-values

**Fig. 1:**
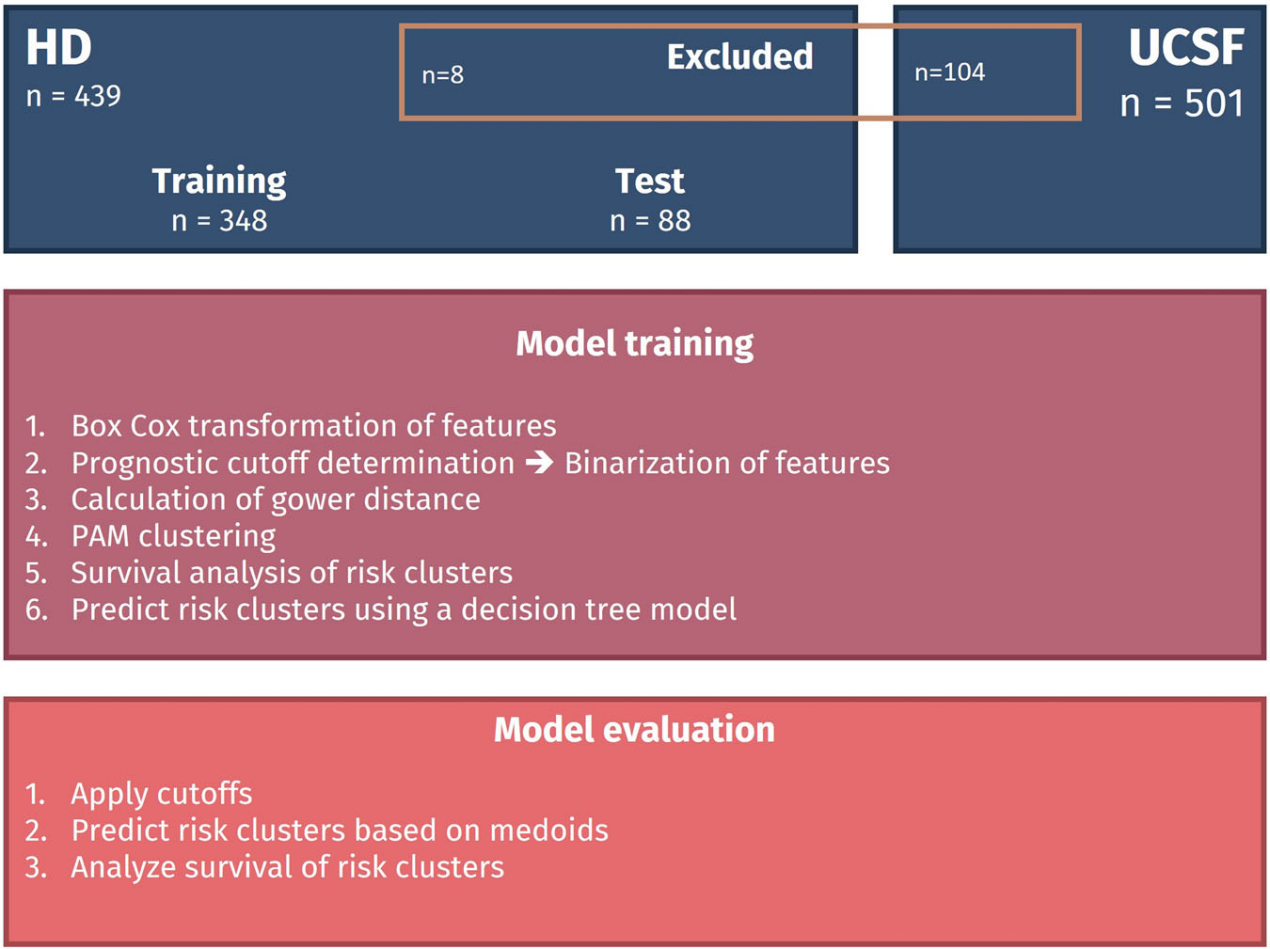
Illustration of the study’s framework. The training set facilitated the determination of thresholds, clustering, and the development of the model. The test dataset served for internal validation, while the UCSF dataset was employed for external assessment of the finalized model

Patients who did not exhibit IDH wild-type status (*n* = 103) or lacked OS data (*n* = 1) were excluded from the UCSF dataset originally comprising *n* = 501 subjects. Consequently, the resultant external test dataset encompassed a total of *n* = 397 (79%) subjects. A graphic representation of the study is shown in Fig. 1. To simplify the prognostic models, we binarized the imaging parameters using the biomarker threshold model in the training dataset. Supplementary Fig. 2 demonstrates the Spearman correlation coefficients among the numerical parameters before binarization (A). The results indicate a strong correlation between several shape features and tumor volume. Panel B, presents the phi correlation coefficients for the binarized parameters. The correlation values generally decrease through binarization.

### Clustering results

PAM clustering revealed the optimal number of two clusters based on the highest average silhouette coefficient (width = 0.44) with high stability (mean Jaccard index of 0.94 for cluster 1 and 0.89 for cluster 2).

**Fig. 2:**
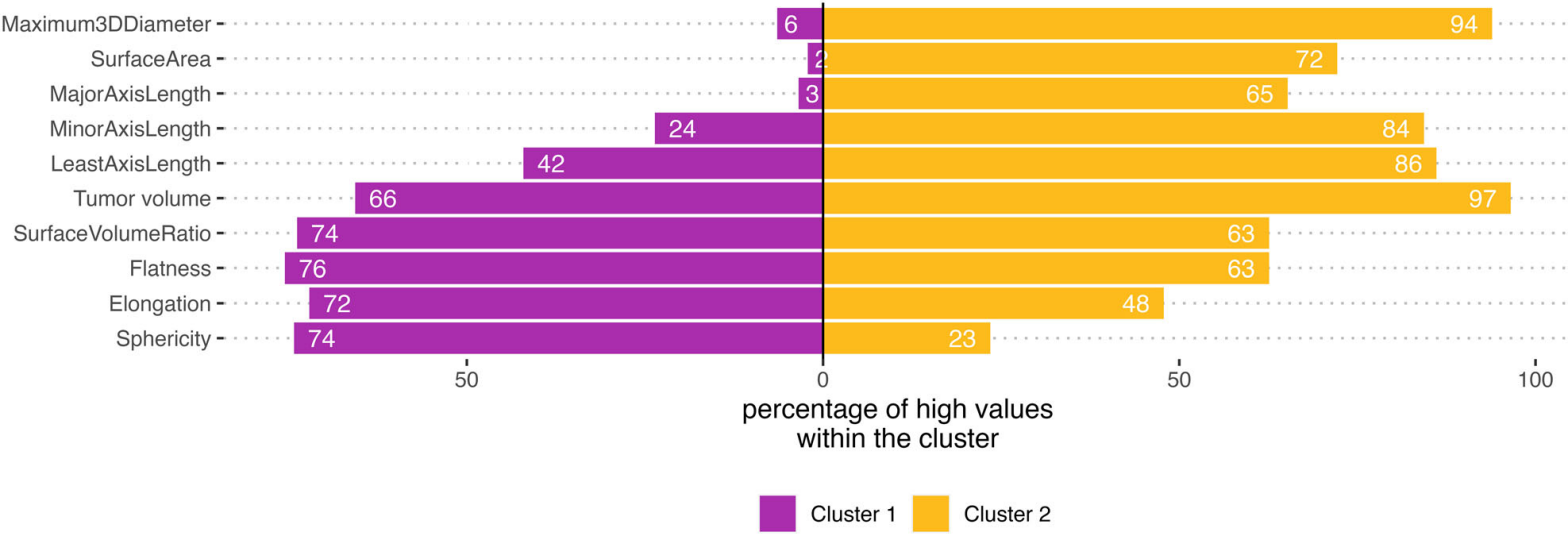
Percentages of high imaging parameters within the two clusters in the training dataset (*n* = 348). Cluster 1 (*n* = 233) shows a significantly higher proportion of patients with high sphericity and elongation compared to cluster 2 (*n* = 115). On the other hand, cluster 2 showed a significantly higher proportion of patients with high maximum 3D diameter, surface area, minor axis length, major axis length, and least axis length (*p*-value < 0.001 each)

Composition analysis of the clusters reveals different patterns of the clusters. Thus, showed cluster 1 (*n* = 233, 67%) a significantly higher proportion of patients with high sphericity and elongation compared to cluster 2 (*n* = 115, 33%) (*p*-value < 0.001 each). On the other hand, cluster 2 showed a significantly higher proportion of patients with high maximum 3D diameter, surface area, minor axis length, major axis length, and least axis length (*p*-value < 0.001 each). There was no significant difference between the clusters in surface volume ratio and flatness Fig. 2.

**Fig. 3:**
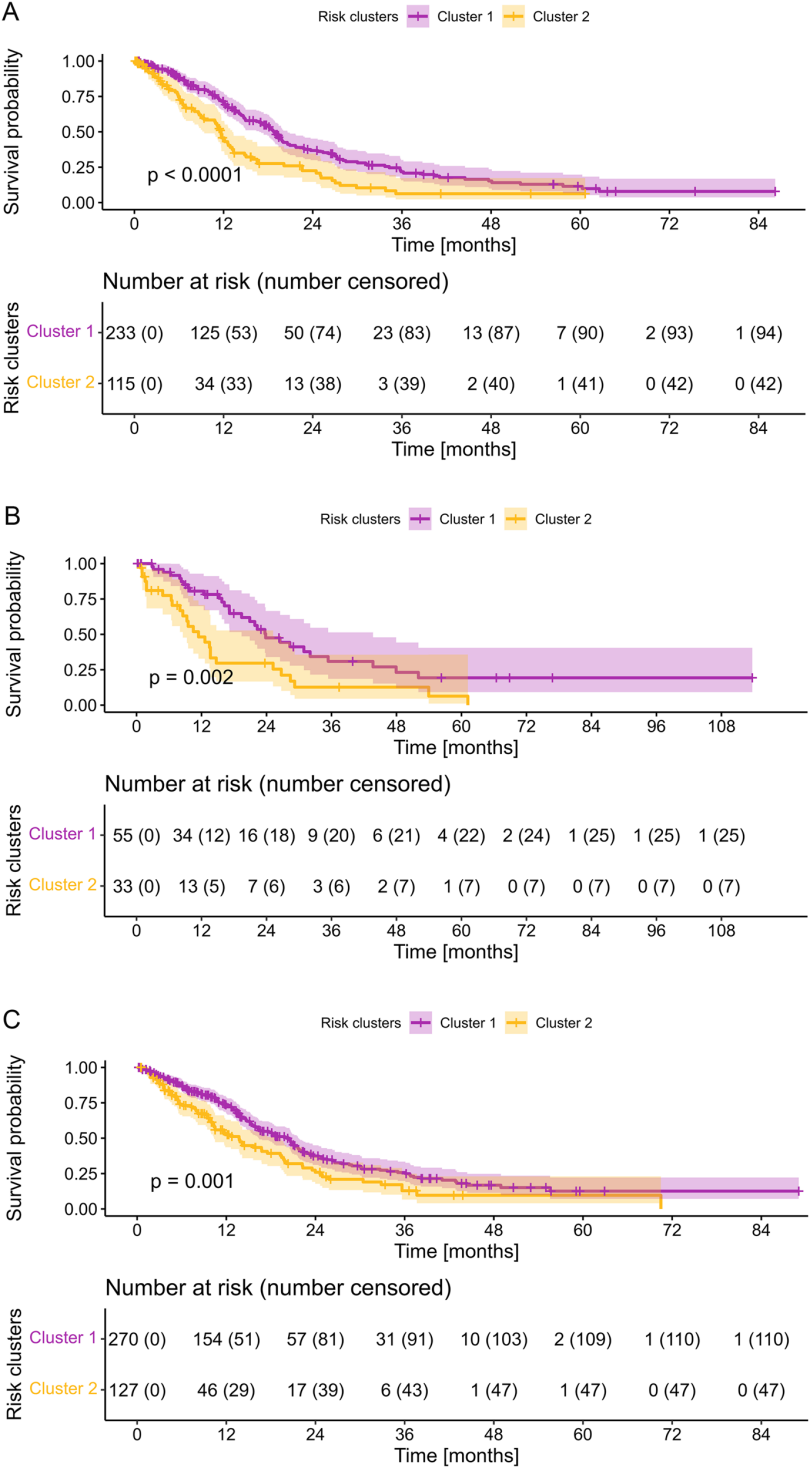
Kaplan–Meier plots of OS in the training dataset (**A**), test dataset (**B**), and UCSF dataset (**C**), with corresponding shaded areas indicating the 95% CIs, stratified to low or high-risk groups according to clustering. Significance was calculated using the log-rank test

Both clusters exhibited a notable contrast in OS, with cluster 1 demonstrating a median OS of 18.8 months (95% confidence interval (CI): 16.3–20.6) and cluster 2 showing a median OS of 11.7 months (95% CI: 9.4–13.2) in the training dataset (log-rank test *p* < 0.001). This trend persisted in the test dataset, with cluster 1 displaying a median OS of 23.8 months (95% CI: 19.4–43.7) compared to 11.4 months (95% CI: 8.6–25.2) for cluster 2 (log-rank test *p* = 0.002). Similarly, in the UCSF dataset, cluster 1 exhibited a median OS of 20.1 months (95% CI: 16.4–21.7) while cluster 2 showed a median OS of 13.7 months (95% CI: 10.4–19.3) (log-rank test *p* = 0.001) (Fig. 3).

In the test dataset, univariate Cox regression analysis illustrated a significant association between tumor volume and OS, with a hazard ratio (HR) of 1 (95% CI: 1.00–1.01) and *p*-value of 0.03, yielding a *C* index of 0.627. Additionally, the analysis revealed a significant association between cluster affiliation and OS, with an HR of 2.25 (95% CI: 1.32–3.84) and a *p*-value of 0.003, yielding a *C* index of 0.625. Moreover, multivariate Cox regression analysis demonstrated that incorporating cluster affiliation into the model improved its performance, elevating the *C* index from 0.59 to 0.67 compared to a model solely based on clinical data (age and sex) (*p* = 0.003). Furthermore, the inclusion of preoperative ECOG status in conjunction with cluster affiliation enhanced the model’s predictive capacity, as evidenced by a higher *C* index of 0.68 compared to 0.61 for the model without cluster affiliation (*p* = 0.005).

When evaluating different model configurations, the model incorporating clinical data and cluster affiliation (AIC = 388.56) outperformed the model based on clinical data and tumor volume (AIC = 391.84), as indicated by an evidence ratio of 5.16. Additionally, the inclusion of preoperative ECOG status further strengthened the superiority of the cluster-based model, with an evidence ratio of 5.37 over the tumor volume model (AIC = 387.95 vs AIC = 391.31, respectively). These findings are summarized in Table 2.

**Table 2:** Performance measures of multivariate Cox proportional hazard models used to predict OS in the test set

Models	Parameter	HR, 95% CI	*p*-value	*C* index	AIC
Model1: cluster (univariate)	Cluster 2	2.25, 1.32–3.84	0.003*	0.625	385.21
Model 2a: cluster + sex + age	Cluster 2 Sex w Age	2.27, 1.33–3.87 1.00, 0.57–1.72 1.01, 0.99–1.04	0.003* 0.973 0.354	0.671	388.56
Model 2b: sex + age	Sex w Age	0.98, 0.56–1.70 1.01, 0.99–1.04	0.943 0.380	0.592	395.09
Model 2c: tumor volume + sex + age	Tumor volume Sex w Age	1.01, 1.00–1.01 1.21, 0.67–2.17 1.02, 0.99–1.04	0.018* 0.527 0.230	0.641	391.84
Model 3a: cluster + sex + age + ECOG	Cluster Sex w Age ECOG ≤ 2	2.20, 1.28–3.78 1.02, 0.58–1.78 1.01, 0.99–1.04 0.09, 0.01–0.80	0.004* 0.947 0.368 0.031*	0.676	387.95
Model 3b: sex + age + ECOG	Sex w Age ECOG ≤ 2	1.01, 0.58–1.76 1.01, 0.99–1.04 0.06, 0.01–0.52	0.971 0.397 0.011*	0.608	393.64
Model 3c: tumor volume + sex + age + ECOG	Tumor volume Sex w Age ECOG ≤ 2	1.01, 1-00–1.01 1.23, 0.68–2.20 1.02, 0.99–1.04 0.09, 0.01–0.85	0.03* 0.497 0.250 0.035*	0.640	391.31

Statistically significant results with a *p*-value < 0.05 are printed in bold and marked with an asterisk

### Explanatory analysis

The tuned decision tree had a tree depth of 3 and a cost complexity of 178 × 10^−4^. Examination of feature importance revealed maximum 3D diameter as the most important prognostic parameter in this model Supplementary Fig. 3.

The structure of the decision tree trained is shown in Supplementary Fig. 4. The tree structure reveals maximum 3D diameter, surface area, and major axis length as the most important features. The validation in the test and UCSF datasets unveiled a high accuracy of 0.91 and 0.96, respectively with a misclassification rate of 9.1% (8 patients) and 4.5% (18 patients).

## Discussion

While high-order radiomic features show promise as prognostic tools for OS prediction in high-grade glioma patients, the potential of shape radiomic features as a prognostic parameter remains underexplored. In this study, we employed a method that avoids predefined classifications or guidance, focusing on identifying survival clusters based on the tumor volume and shape-related radiomic features in newly diagnosed pre-operative IDH-wt glioma. Our results indicate that incorporating these clusters could enhance prognostic accuracy, offering an advantage over relying solely on clinical assessments or models based on tumor volume alone when predicting OS.

Upon scrutinizing the clusters, we observed that cluster 1, associated with longer OS, had a higher percentage of patients with tumors displaying elevated sphericity and elongation values, suggesting a more spherical shape. Conversely, the fraction of patients in this cluster with high values in maximum 3D diameter, surface area, and axis length was markedly lower in comparison to cluster 2. These findings are consistent with the work of Pérez-Betata et al, who demonstrated that surface regularity, derived from contrast-enhanced T1-weighted pre-treatment MRI sequences, serves as a robust indicator of survival in a cohort of 165 GB patients from five local institutions [33]. This parameter quantifies the degree of deviation of the tumor’s surface from that of a sphere with corresponding volume. The study demonstrated that surface regularity serves as a robust indicator of patient survival. Notably, it was observed that patients with higher surface regularity, indicative of tumors with a shape akin to a sphere, exhibited prolonged survival periods [33]. These results are consistent with many other studies, that showed that the irregularity of the tumor margins is a good prognostic parameter for survival in IDH-wt glioma patients [13, 34]. In contrast to Perez-Betata’s focus on the surface area of contrast-enhancing compartments, our study extracted radiomics from the entire tumor including non-contrast enhancing FLAIR-hyperintense parts, suggesting that the shape of these parts also holds prognostic value.

Through different multivariable Cox regression models, we showed that adding cluster membership outperformed clinical and volume-based models alone. Notably, each model indicated the independent significance of cluster membership in association with OS, consistent with numerous studies recognizing preoperative tumors as a reliable prognostic indicator [5, 6, 18]. Furthermore, Sanghani et al emphasized the prognostic significance of shape features for OS prognosis in GB patients [12]. In univariate and multivariate Cox regression analyses, they showed that bounding ellipsoid volume ratio, sphericity, and spherical disproportion, calculated from contrast-enhancing and FLAIR hyperintense not contrast-enhancing tumor parts, are significant for OS prediction. Our correlation analysis demonstrated that shape features such as diameter, surface area, and major axis length correlate with volume. However, they also capture unique aspects of the tumor’s geometry that volume alone does not fully represent. Phi correlation coefficients for the binarized parameters show decreased correlations after binarization. This reduction highlights how binarization mitigates inherent biases due to the scale and magnitude of the original numerical parameters. Consequently, while volume is a significant descriptor, it does not encompass all the nuances of the tumor’s shape. Thus, although tumor volume is correlated with various shape features, the multidimensional aspects represented by shape radiomics provide additional and valuable insights.

Using a decision-tree model we identified maximum 3D diameter as the most important parameter for determining cluster membership. In contrast to the volume, which measures the total size of the tumor, the maximum 3D diameter represents the largest possible length of the tumor measured through the tumor’s center, providing insights into its spatial extent. The comparative prognostic value of maximum 3D diameter relative to tumor volume in IDH-wt glioma remains unexplored, but similar findings in lung cancer suggest a potential link to the characteristic nature of tumors with irregular shapes impacting patient outcomes more than total volume [35].

While our study provides valuable insights, it is important to note some limitations: First, this study is retrospective and trained with a monocentric data set, the clustering may be influenced by specific characteristics of the study population. However, we used an internal test dataset and an external, publicly available, dataset for further validation. Second, apart from IDH status, we did not consider any other molecular markers, such as TERT promoter status. As such, we cannot comment on whether the prognostic role of shape or the natural disease course varies across molecular IDH-wt variants defined by their molecular or epigenetic profile as previously postulated [36]. Future studies should therefore focus on the relationship between IDH-wt shape and other molecular markers.

In conclusion, our data-driven, unsupervised technique identified distinct and clinically relevant imaging phenotypes through shape radiomics, enhancing the ability to predict survival outcomes in newly diagnosed high-grade glioma patients. This approach can improve the capability to predict survival outcomes in high-grade glioma patients, underscoring its potential value in clinical settings.

## Supplementary information

ELECTRONIC SUPPLEMENTARY MATERIAL

## Abbreviations

AIC: Akaike information criterion
IDH-wt: Isocitrate dehydrogenase-wildtype
PAM: Partition around medoids
RANO: Response assessment in neuro-oncology
UCSF: University of California San Francisco
