# Supplementary material for: Shape matters: unsupervised exploration of IDH-wildtype glioma imaging survival predictors
Source: Eur Radiol. 2024 Sep 9;35(3):1351–60. doi: 10.1007/s00330-024-11042-6 (PMC11835892; doi:10.1007/s00330-024-11042-6)

Shape Matters: Unsupervised Exploration of IDH-wildtype Glioma Imaging Survival Predictors

ELECTRONIC SUPPLEMENTARY MATERIAL

Supplementary Materials and Methods

The following features are based on the estimated form created by the triangular mesh. This mesh is constructed by initially identifying vertices, which are points located midway along an edge between a voxel inside the Region of Interest (ROI) and a voxel outside it. These vertices are then linked to form a mesh composed of interconnected triangles. Each triangle in the mesh is determined by three neighboring vertices and shares each of its sides with exactly one other triangle. A more detailed description can be found on the PyRadiomics homepage (https://pyradiomics.readthedocs.io/en/latest/index.html).

Maximum 3D Diameter: Maximum 3D Diameter represents the largest pairwise euclidean distance between tumor surface mesh vertices.

Surface Area: The Surface Area measures the outer boundary of the tumor. It is the sum of the Surface Areas of the individual triangles in the mesh. In contrast to volume Surface Area is more related to the external shape and contours of the tumor.

Surface Volume Ratio: Represents the ratio between the Surface Area and volume of the tumor. A smaller value suggests a more compact shape, like a sphere. Since this feature is not dimensionless it is (partly) dependent on the volume of the tumor.

Major Axis Length: Represents the largest axis length of the tumor-enclosing ellipsoid.

Minor Axis Length: Represents the second-largest axis length of the tumor-enclosing ellipsoid.

Least Axis Length: Represents the smallest axis length of the tumor-enclosing ellipsoid.

Sphericity: Sphericity quantifies how closely the tumor region's shape resembles a sphere. It's a scale-independent and orientation-neutral metric without dimensions. The sphericity values range from 0 to 1, with 1 signifying an ideal sphere. A sphere is unique as it has a minimal surface area for a given volume compared to other shapes.

Elongation: Elongation illustrates the ratio of the two principal components with the greatest size in the shape of the tumor. For practical computational purposes, this feature is calculated as the inverse of the actual elongation measure. The values of elongation range from 1, which indicates a shape where the cross-section through the first and second largest principal components resembles a circle (indicating it is not elongated), to 0, which indicates an extremely elongated shape, akin to a one-dimensional line. This measurement is derived from principal component analysis, which is conducted using the physical coordinates of the voxel centers that define the tumor. This analysis accounts for the spacing between voxels but does not incorporate the shape mesh.

Flatness: Flatness measures the ratio between the smallest and the largest principal components in the shape of the tumor. For ease of computation, this attribute is determined as the inverse of the actual flatness value. The flatness values vary from 1, indicating a non-flat, sphere-like shape, to 0, which represents an extremely flat object.

In addition to the tumor volume using FSL, we also extracted the Mesh Volume as a radiomic feature from pyradiomics.

Mesh Volume: The volume of the ROI is determined using a triangle mesh. For each triangle in this mesh, the volume of the tetrahedron created by the triangle and the origin point of the image is calculated.

We conducted a Pearson correlation analysis to compare the whole tumor volume measurements obtained from FSL and the Mesh Volume feature extracted from PyRadiomics.

Supplementary CLEAR Checklist

CLEAR checklist without explanations

Section	No.	Item	Yes	No	n/a
Title					
	1	Relevant title, specifying the radiomic methodology	X	☐	☐
Abstract					
	2	Structured summary with relevant information	X	☐	☐
Keywords					
	3	Relevant keywords for radiomics	X	☐	☐
Introduction					
	4	Scientific or clinical background	X	☐	☐
	5	Rationale for using a radiomic approach	X	☐	☐
	6	Study objective(s)	X	☐	☐
Method					
Study Design	7	Adherence to guidelines or checklists (e.g., CLEAR checklist)	X	☐	☐
	8	Ethical details (e.g., approval, consent, data protection)	X	☐	☐
	9	Sample size calculation	☐	☐	X
	10	Study nature (e.g., retrospective, prospective)	X	☐	☐
	11	Eligibility criteria	X	☐	☐
	12	Flowchart for technical pipeline	X	☐	☐
Data	13	Data source (e.g., private, public)	X	☐	☐
	14	Data overlap	X	☐	☐
	15	Data split methodology	X	☐	☐
	16	Imaging protocol (i.e., image acquisition and processing)	X	☐	☐
	17	Definition of non-radiomic predictor variables	X	☐	☐
	18	Definition of the reference standard (i.e., outcome variable)	X	☐	☐
Segmentation	19	Segmentation strategy	X	☐	☐
	20	Details of operators performing segmentation	X	☐	☐
Pre-processing	21	Image pre-processing details	X	☐	☐
	22	Resampling method and its parameters	X	☐	☐
	23	Discretization method and its parameters	X	☐	☐
	24	Image types (e.g., original, filtered, transformed)	X	☐	☐
Feature extraction	25	Feature extraction method	X	☐	☐
	26	Feature classes	X	☐	☐
	27	Number of features	X	☐	☐
	28	Default configuration statement for remaining parameters	X	☐	☐
Data preparation	29	Handling of missing data	☐	☐	X
	30	Details of class imbalance	☐	☐	X
	31	Details of segmentation reliability analysis	X	☐	☐
	32	Feature scaling details (e.g., normalization, standardization)	X	☐	☐
	33	Dimension reduction details	X	☐	☐
Modeling	34	Algorithm details	X	☐	☐

	35	Training and tuning details	X	☐	☐
	36	Handling of confounders	☐	☐	X
	37	Model selection strategy	X	☐	☐
Evaluation	38	Testing technique (e.g., internal, external)	X	☐	☐
	39	Performance metrics and rationale for choosing	X	☐	☐
	40	Uncertainty evaluation and measures (e.g., confidence intervals)	X	☐	☐
	41	Statistical performance comparison (e.g., DeLong's test)	☐	☐	X
	42	Comparison with non-radiomic and combined methods	X	☐	☐
	43	Interpretability and explainability methods	X	☐	☐
Results					
	44	Baseline demographic and clinical characteristics	X	☐	☐
	45	Flowchart for eligibility criteria	☐	☐	X
	46	Feature statistics (e.g., reproducibility, feature selection)	☐	☐	X
	47	Model performance evaluation	X	☐	☐
	48	Comparison with non-radiomic and combined approaches	X	☐	☐
Discussion					
	49	Overview of important findings	X	☐	☐
	50	Previous works with differences from the current study	X	☐	☐
	51	Practical implications	X	☐	☐
	52	Strengths and limitations (e.g., bias and generalizability issues)	X	☐	☐
Open Science					
Data availability	53	Sharing images along with segmentation data [n/e]	☐	☐	☐
	54	Sharing radiomic feature data	☐	X	☐
Code availability	55	Sharing pre-processing scripts or settings	X	☐	☐
	56	Sharing source code for modeling	X	☐	☐
Model availability	57	Sharing final model files	X	☐	☐
	58	Sharing a ready-to-use system [n/e]	☐	☐	☐

Yes, details provided; **No**, details not provided; **n/e**, not essential; **n/a**, not applicable

Note: Use the checklist in conjunction with the main text for clarification of all items. Fill the "Page" column with the related page number where the information is provided.

Supplementary Results

The correlation coefficient between the tumor volume measurements obtained from FSL and the Mesh Volume feature extracted from PyRadiomics was 1 for the HD and UCSF dataset, indicating an almost perfect linear relationship. This suggests that the volumes measured by both methods are highly consistent.

Supplementary Tables

Supplementary Table 1 Summary of mean and standard deviation of shape radiomics in the HD and UCSF datasets. The p-values indicate the statistical significance of the differences between the two datasets, with values less than 0.001 bolded.

**Parameter**		**HD**	**UCSF**	**p-value**
Surface Area	Mean ± std.	17237± 9623	17430 ± 9757	0.96
Surface Volume Ratio	Mean ± std.	0.25 ± 0.094	0.22 ±0.085	**<0.001**
Sphericity	Mean ± std.	0.54 ± 0.11	0.57 ± 0.11	**<0.001**
Maximum 3D Diameter	Mean ± std.	85 ± 25	91 ± 26	0.05
Major Axis Length	Mean ± std.	71 ± 24	72 ± 22	0.53
Minor Axis Length	Mean ± std.	50 ± 14	51 ± 13	0.47
Least Axis Length	Mean ± std.	38 ± 11	39 ± 11	0.30
Elongation	Mean ± std.	0.73 ± 0.15	0.72 ± 0.14	0.13
Flatness	Mean ± std.	0.57 ± 0.14	0.56 ± 0.13	0.46

Supplementary Table 2 Comprehensive summary of cut-off values for each parameter. The table provides the original cut-off value, the box-cox transformed cut-off value, and the corresponding lambda value used in the box-cox transformation.

**Parameter**	**Cut-off value**	**Box-Cox cut-off value**	**Lambda**
Surface Area	23915	509.69	0.56
Surface Volume Ratio	0.19	-4.45	-1.01
Sphericity	0.51	-0.51	0.85
Maximum 3D Diameter	96.13	219.32	1.23
Major Axis Length	85.78	23.48	0.61
Minor Axis Length	53.39	60.52	1.05
Least Axis Length	37.11	58.61	1.18
Elongation	0.70	-0.24	2.27
Flatness	0.50	-0.43	1.54
Whole tumor volume	34.63	8.35	0.43

Supplementary Table 3 R Packages and Versions

Package	Version
writexl	1.4.2
ggcorrplot	0.1.4.1
DescTools	0.99.47
AICcmodavg	2.3-2
cowplot	1.1.1
corrplot	0.92
psych	2.2.9
doParallel	1.0.17
iterators	1.0.14
foreach	1.5.2
baguette	1.0.1
vip	0.3.2
rpart.plot	3.1.1
DataExplorer	0.8.2
UpSetR_	1.4.0
survminer	0.4.9
factoextra	1.0.7
cluster	2.1.4

bhm	1.18
survival	3.5-0
MASS	7.3-58.2
naniar	1.0.0
kableExtra	1.3.4
ggpubr	0.6.0
DescrTab2	2.1.16
here	1.0.1
yardstick	1.2.0
tidymodels	1.0.0
dplyr	1.1.1
purrr	1.0.1
tidyr	1.3.0
tibble	3.2.1
ggplot2	3.4.2
tidyverse	1.3.2
readxl	1.4.2

Supplementary Figures

Supplementary Figure 1 Kaplan-Meier plot of OS between training- and test data set. Significance was calculated using a log-rank test.

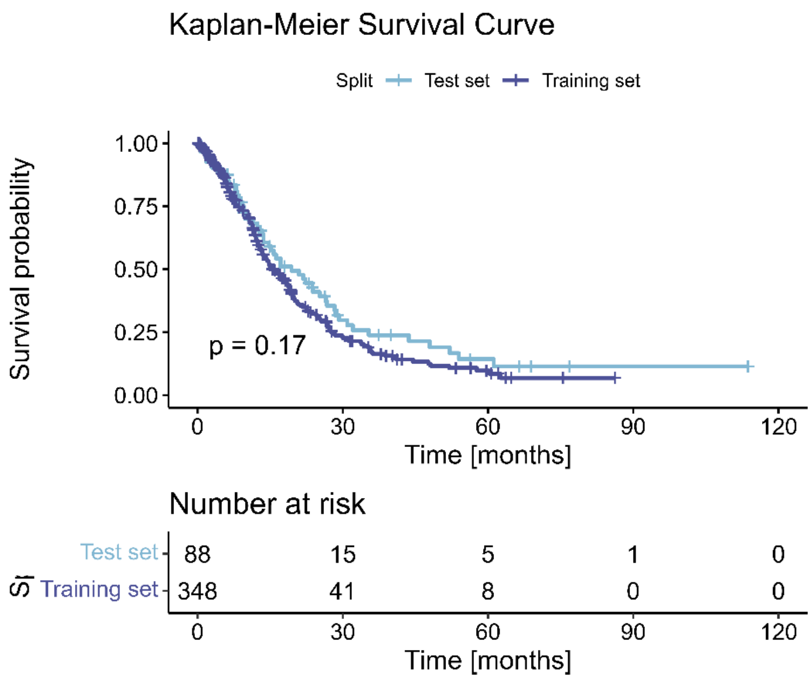

Supplementary Figure 2 Spearman correlation of the numerical parameters before binarization (A) and phi correlation of the different binarized parameters (B) in the training data

A

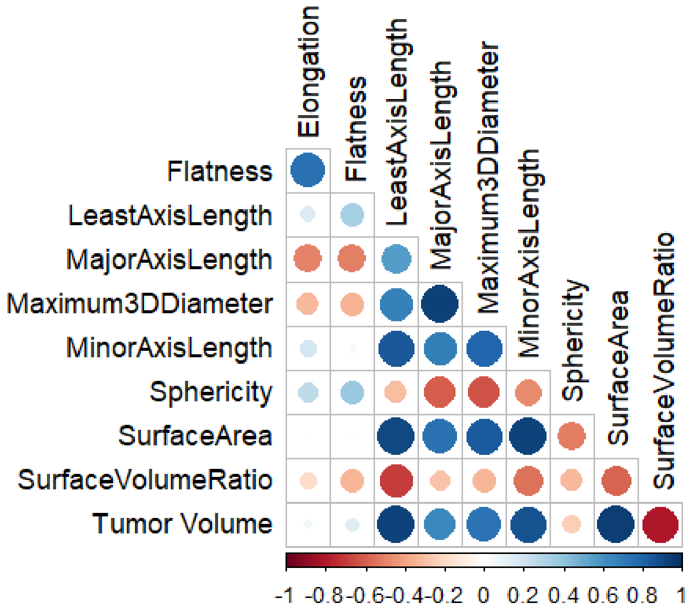

set. +1 indicates perfect agreement and -1 perfect disagreement.

B

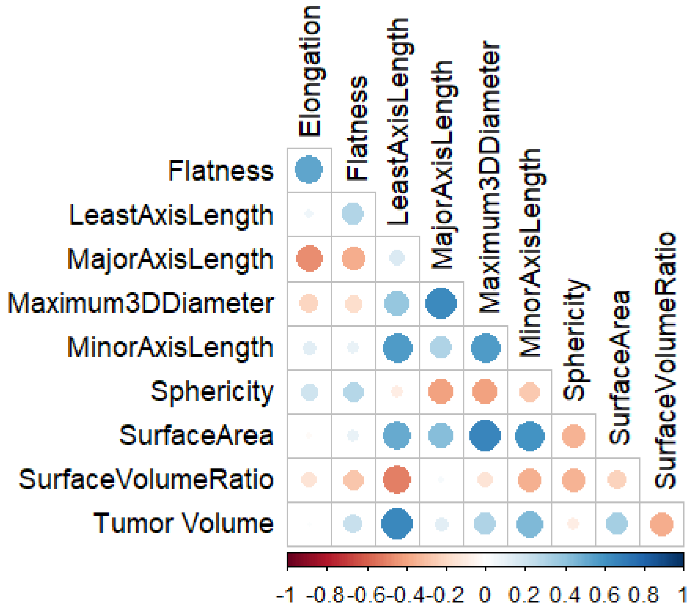

Supplementary Figure 3 Horizontal bar chart of feature importance according to the decision tree model.

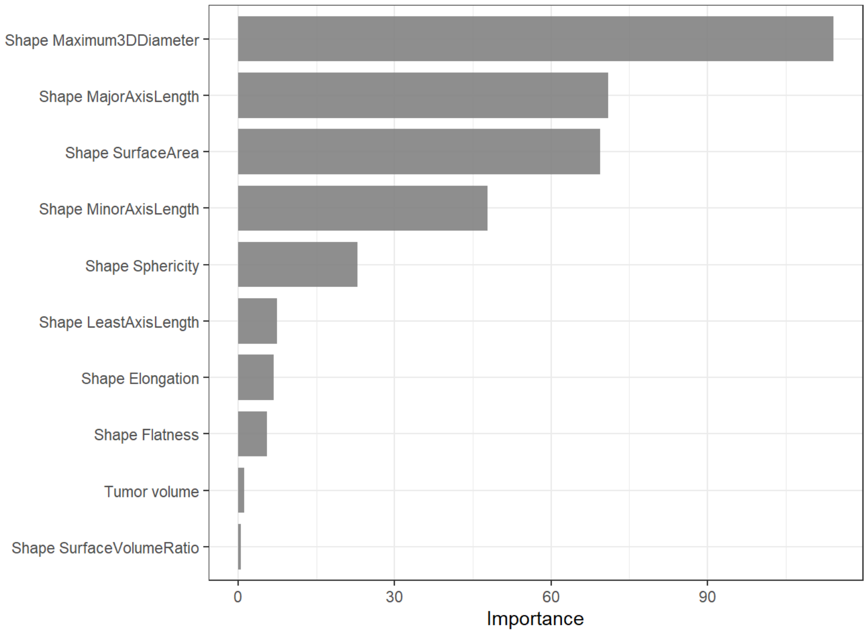

Supplementary Figure 4 Structure of the trained decision tree with the training data set. The first number in the leaf shows the cluster membership, the second the probability for this cluster, and the third the percentage of patients in the dataset who have been classified into this leaf.

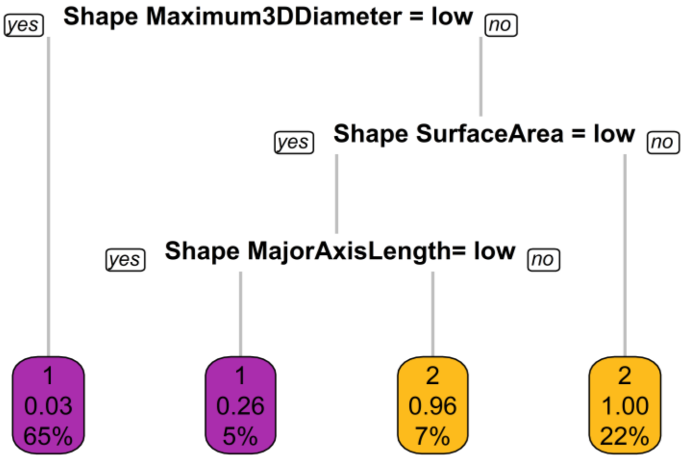

Supplement: Supplementary file 1 — ELECTRONIC SUPPLEMENTARY MATERIAL [file 330_2024_11042_MOESM1_ESM.pdf]
